# Inhibition by Commercial Aminoglycosides of Human Connexin Hemichannels Expressed in Bacteria

**DOI:** 10.3390/molecules22122063

**Published:** 2017-11-25

**Authors:** Mariana C. Fiori, Srinivasan Krishnan, Abbey Kjellgren, Luis G. Cuello, Guillermo A. Altenberg

**Affiliations:** 1 Department of Cell Physiology and Molecular Biophysics, and Center for Membrane Protein Research, Texas Tech University Health Sciences Center, Lubbock, TX 79430-6551, USA; mariana.fiori@ttuhsc.edu (M.C.F.); srinivas.krishnan21@gmail.com (S.K.); abbey.kjellgren@ttu.edu (A.K.); luis.cuello@ttuhsc.edu (L.G.C.); 2 Honors College, McClellan Hall, Box 41017, Texas Tech University, Lubbock, TX 79409-1017, USA

**Keywords:** gap junction, channel, Cx26, Cx43, Cx46, screening, LB2003, antibiotic, cell-based assay, transport

## Abstract

In addition to gap junctional channels that mediate cell-to-cell communication, connexins form hemichannels that are present at the plasma membrane. Since hemichannels are permeable to small hydrophilic compounds, including metabolites and signaling molecules, their abnormal opening can cause or contribute to cell damage in disorders such as cardiac infarct, stroke, deafness, skin diseases, and cataracts. Therefore, hemichannels are potential pharmacological targets. A few aminoglycosides, well-known broad-spectrum antibiotics, have been shown to inhibit hemichannels. Here, we tested several commercially available aminoglycosides for inhibition of human connexin hemichannels using a cell-based bacterial growth complementation assay that we developed recently. We found that kanamycin A, kanamycin B, geneticin, neomycin, and paromomycin are effective inhibitors of hemichannels formed by connexins 26, 43, and 46 (Cx26, Cx43, and Cx46). Because of the >70 years of clinical experience with aminoglycosides and the fact that several of the aminoglycosides tested here have been used in humans, they are promising starting points for the development of effective connexin hemichannel inhibitors.

## 1. Introduction

**Figure 1:**
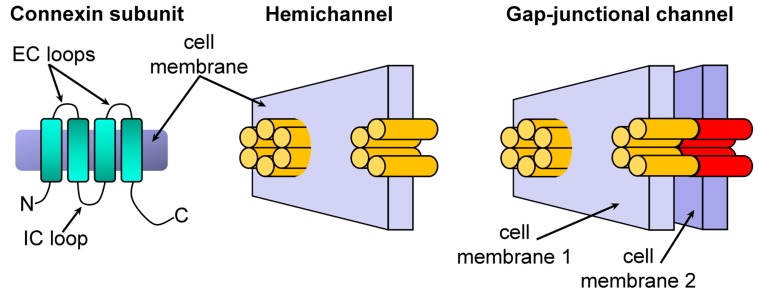
Connexins, gap-junction channels, and hemichannels. Schematic representation of a connexin subunit (monomer), an hemichannel (HC) formed by six connexins, and a gap-junctional channel (GJC) formed by two HCs docked head to head. The cyan rounded rectangles in the connexin denote transmembrane helices. EC: extracellular; IC: intracellular. In the HCs and GJC each subunit is illustrated as a cylinder. Adapted from reference [14]; reproduced with permission from the American Society for Biochemistry and Molecular Biology.

Gap-junction channels (GJCs) and hemichannels (HCs) are connexin oligomers [1,2]. HCs are connexin hexamers, whereas GJCs are formed by docking of two HCs head-to-head (Figure 1). Each of the two HCs of a GJC originates from a different adjacent cell; i.e., each cell contributes one HC to the GJC. The role of GJCs in cell-to-cell communication in health and disease has been known for several decades, but demonstration of undocked, free HCs in the plasma membrane and their role in physiological and pathophysiological processes is more recent [3,4]. GJCs and HCs contain a hydrophilic permeation pathway for molecules of up to 600–800 Da, depending on the isoform [2,5]. Whereas GJCs couple neighboring cells electrically by virtue of their poorly selective permeability to small inorganic ions such as K^+^ , Cl^−^, and Na^+^ [5], HCs (which are mostly closed) are involved in autocrine and paracrine signaling by mediating the regulated efflux of adenosine triphosphate (ATP), nicotinamide adenine dinucleotide (NAD^+^), glutamate, prostaglandins, and other molecules from cells [6]. HCs have also been implicated in the pathophysiology of important disorders such as deafness, cataracts, and ischemic damage of the heart, brain, and kidneys [7,8,9,10,11,12]. Abnormal opening of the non-selective “large” HCs can cause or contribute to cell damage by causing depolarization, cell swelling, and alterations in cellular electrolytes, metabolites, and second messengers. Therefore, connexin HCs are relevant pharmacological targets and inhibitors can be useful for treatment of disorders associated with sustained HC opening (e.g., ischemic disorders) [4,10,11,13].

Hemichannel inhibitors used in research are many and structurally diverse, including carbenoxolone, 2-aminoethoxydiphenyl borate, antimalarial drugs, 18-β-glycyrrhetinic acid, and octanol [15,16,17]. These compounds are not appropriate starting points for development of inhibitors for clinical use because they are not selective for connexin HCs, and their toxicity is in many cases an important issue. The case of connexin peptide inhibitors is different. They are short synthetic peptides corresponding to the sequences of extracellular or intracellular loops of connexins. It is thought that the former (e.g., ^43^Gap26, ^43^Gap27, ^40^Gap27 and peptide 5) associate with HC extracellular loops, preventing docking and formation of GJCs, whereas the latter act by disrupting interactions between the intracellular loop and C-terminal region. Most inhibit GJCs and HCs, but a few that target extracellular lops (e.g., ^37^Gap26, peptide 5) or the intracellular loop (e.g., Gap19, Gap24) have been reported to inhibit only HCs or to display significant HC/GJC selectivity [18]. The rational design of connexin-targeted peptide inhibitors is promising, and such peptides have been used to treat arrhythmias and to accelerate wound healing [19,20]. However, their potential as useful clinical agents is still unclear, and in any case, other avenues to identify clinically useful HC inhibitors have been largely unexplored.

**Figure 2:**
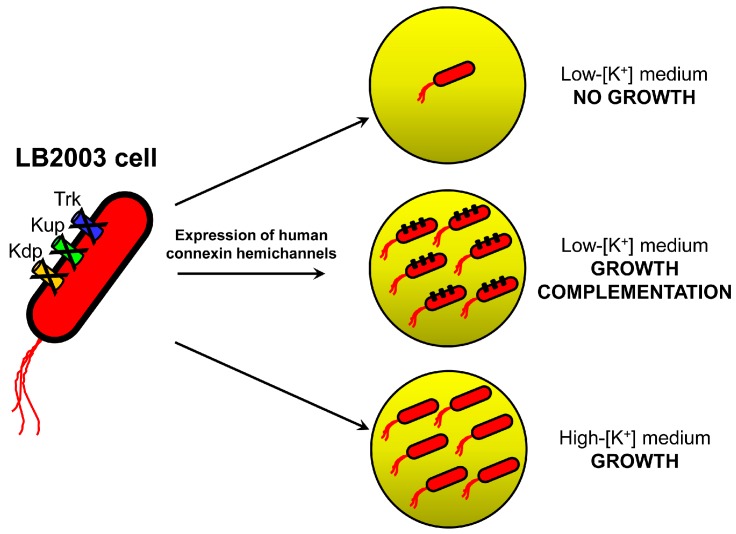
Schematic representation of the growth complementation assay used to assess the function of recombinant human hemichannels expressed in LB2003 cells. Adapted from reference [22] with permission from SAGE Publications.

Recently, we found that human connexin 26 (Cx26) and connexin 43 (Cx43) HCs can be functionally expressed in *Escherichia coli*, and we developed a growth-complementation assay to assess HC function using LB2003 as host [21,22]. LB2003 *E. coli* cells are missing three K^+^ uptake systems and cannot grow in low-[K^+^] medium [22,23,24]. However, their inability to grow in low-[K^+^] medium can be rescued by expression of recombinant K^+^-selective channels or connexin HCs (Figure 2) [22,23,25,26]. Expression of the “large” poorly selective HCs results in increased K^+^ permeability and influx, which allows growth in low-[K^+^] media; K^+^ is required for maintenance of turgor pressure, enzyme activation, intracellular pH regulation, etc.

In previous reports, it was found that the aminoglycoside (AG) gentamicin does not affect GJCs, but inhibits HCs reversibly in mammalian cells and frog oocytes [27,28]. Using our simple, inexpensive and robust assay based on LB2003 cells growth, we found that kanamycin A, another AG, also inhibited HCs [21,22]. AGs are a structurally diverse group of broad-spectrum antibiotics that contain various numbers of normal and unusual sugars [29,30]. Nephrotoxicity and ototoxicity are relatively common complications of the treatment with AGs, but they can be managed [31], and AGs are still among the most used antibiotics worldwide. Since several AGs are approved for use in humans and are well-known from >70 years of clinical use, they are potentially good starting points for the development of effective HC inhibitors. Here, we aimed at testing several commercially available AGs to determine their inhibitory effect on bacterial growth complementation dependent on Cx26, Cx43, and Cx46, with the idea of identifying HC compounds that can serve as starting points for the design and synthesis of new inhibitors.

## 2. Results and Discussion

### 2.1. Growth Complementation by Cx26, Cx43 and Cx46

**Figure 3:**
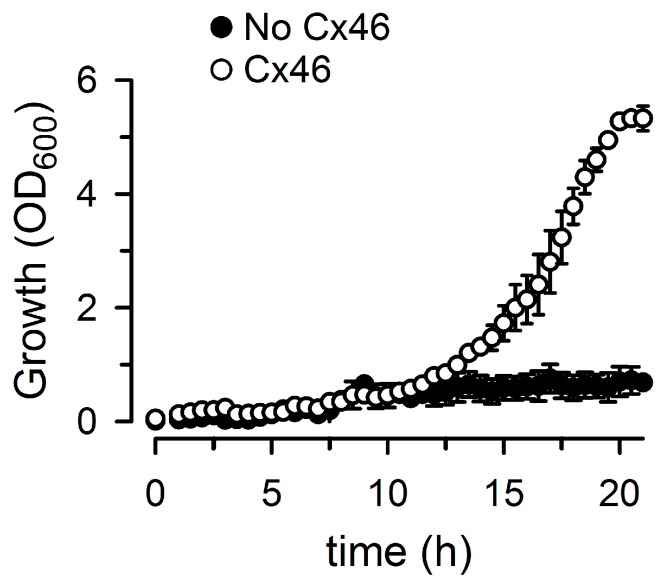
Growth complementation by Cx46. Time course of the growth complementation by Cx46. LB2003 cells were grown at 30 °C in Na^+^ liquid medium (NLM) with 16 mM K^+^ and 10 μM isopropyl-β-d-thiogalactopyranoside (IPTG). No Cx46: cells transformed with the empty plasmid pQE60; Cx46: cells transformed with the human Cx46 into pQE60. OD_600_: absorbance measured at 600 nm. Data are means ± standard error (SEM) from three independent experiments. SEM smaller than the size of the symbols are not shown.

Hemichannels formed by Cx26, Cx43, and Cx46 are present in a variety of tissues and organs. Cx26 is associated with the physiology and pathophysiology of the inner ear, Cx43 with that of heart, brain, and kidneys, and Cx46 with that of the lens [7,8,9,10,32]. We have previously determined the best conditions for the growth complementation assay associated with the expression of Cx26 and Cx43 [22]. Based on our experience with these connexins, the most important tasks for the optimization of the assay are to increase reproducibility and the signal-to-noise ratio. The first task is accomplished through the use of defined media. We use Na^+^ liquid medium (NLM) and K^+^ liquid medium (KLM). NLM is a K^+^-free phosphate-buffered medium (see Materials and Methods), whereas KLM is a K^+^-rich medium that has the composition of NLM, except for the equimolar replacement of Na^+^ with K^+^. LB2003 cells do not grow in NLM, but grow in KLM [22]. By mixing NLM and KLM it is easy to find a range of low [K^+^]s where LB2003 cells expressing connexins grow. To optimize the signal-to-noise ratio we identified the “low” [K^+^] at which the connexin-expressing cells grow to the highest absorbance measured at 600 nm (OD_600_), and then tested the dependence of the growth on the concentration of the expression inducer, isopropyl-β-d-thiogalactopyranoside (IPTG). This is important because the optimal signal-to-noise ratio depends on a balance between sufficient permeability to allow K^+^ uptake through HCs and too many active HCs, which inhibits growth as a result of depolarization of the inner membrane and/or unbalanced shifts of ions and metabolites. In the case of Cx26, optimal growth is achieved with 4 mM K^+^ and 500 μM IPTG, but for Cx43, 8 mM K^+^ and 10 μM IPTG work best. From initial trials, we identified 16 mM K^+^ and 10 μM IPTG as the best conditions for the Cx46 growth complementation assay. Figure 3 illustrates the time dependence of the growth of LB2003 cells expressing Cx46 and shows that human Cx46 can complement the growth of LB2003 cells in low-[K^+^] medium, as Cx26 and Cx43 do.

### 2.2. Inhibition of Cx26-, Cx43- and Cx46-Dependent Growth Complementation by AGs

**Figure 4:**
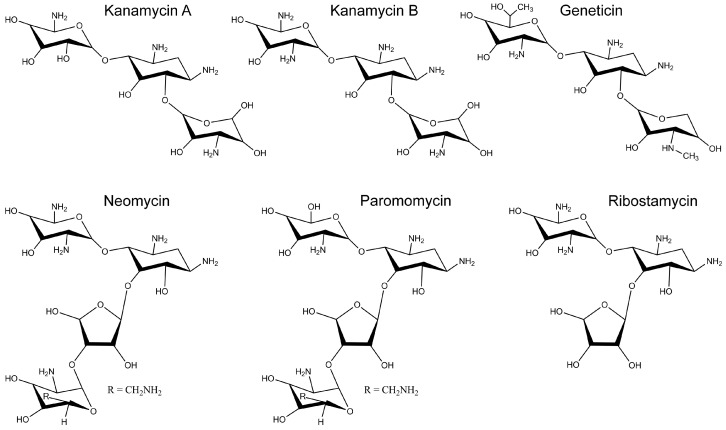
Structures of the aminoglycosides used in this study.

Here, we studied several commercially available AGs that have been used in humans (Figure 4). 

**Figure 5:**
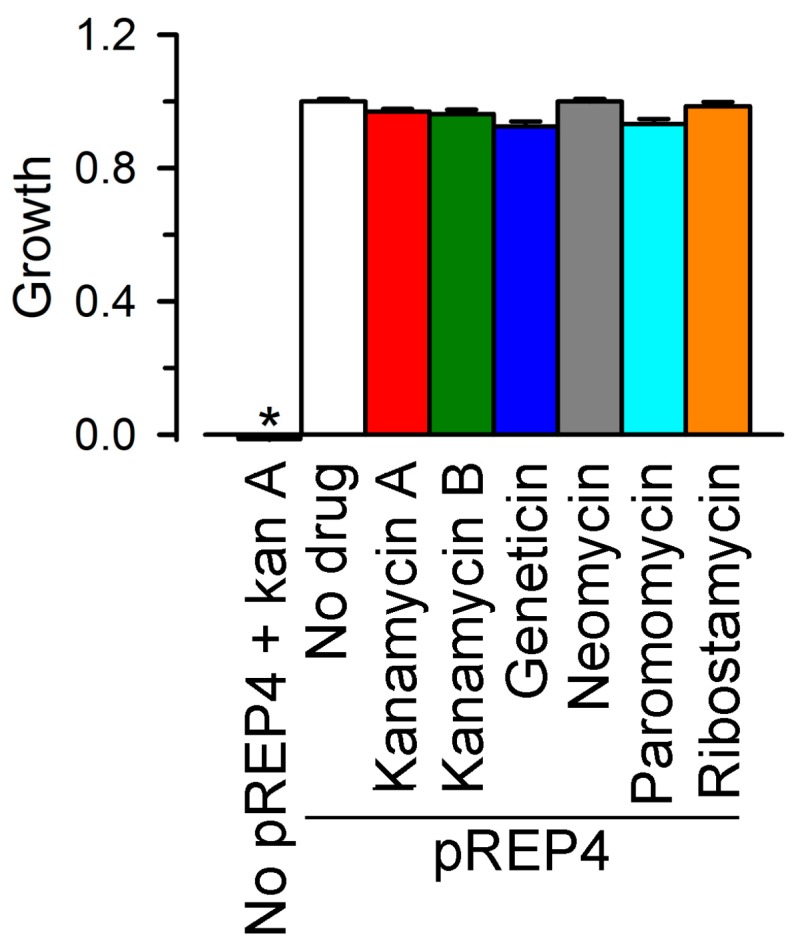
Absence of aminoglycoside (AG) antibiotic effect in LB2003 cells transformed with the pREP4 plasmid. All drugs were used at a concentration of 100 μM. The cells were grown in K^+^ liquid medium (KLM). The pREP4 plasmid carries the neomycin phosphotransferase gene. No pREP4 + kan A: effect of kanamycin A in AG-sensitive cells; all other bars correspond to AG-resistant cells containing the pREP4 plasmid, in the absence (no drug) or presence of the indicated AGs. Data were normalized to the OD_600_ of LB2003-pREP4 cells in the absence of drug (5.7 ± 0.1). Data are means ± SEM of three independent experiments (in triplicate) per condition. * denotes a *p* < 0.001 vs. all other groups.

For these studies, LB2003 cells were transformed with pREP4, a plasmid that carries the neomycin phosphotransferase gene. This aminoglycoside 3'-phosphotransferase inactivates the AGs in Figure 4 by phosphorylation, allowing us to test for inhibition of connexin-dependent growth complementation without interference by the AG antibiotic effect. The absence of antibiotic effect in the LB2003-pREP4 cells was assayed at an AG concentration of 100 μM in cells grown in KLM (Figure 5). The figure shows that, in LB2003 cells sensitive to the AGs (no pREP4), kanamycin prevented growth (pREP4 + kan A bar), whereas growth of the AG-resistant LB2003 cells containing the pREP4 plasmid was not affected by the AGs, with relative growth similar to that in the absence of drug. We then tested all AGs in Figure 4 for inhibition of connexin-dependent growth in AG-resistant LB2003 cells transformed with the pREP4 plasmid. Except for ribostamycin, which showed only a marginal effect, all AGs produced a significant inhibition of connexin-dependent growth at a concentration of 100 μM (see next section). At a concentration of 100 μM, ribostamycin reduced Cx26-dependent growth complementation by only 35 ± 3% (*n* = 3, *p* < 0.01) and had no effect on Cx43-dependent growth complementation (−8 ± 5%; *n* = 3). For comparison, the inhibition of Cx26-dependent growth complementation by 100 μM paromomycin was ~80% and that by the other AGs was >90% (see below). Based on these observations, we follow up with concentration-dependence studies of the inhibition by kanamycin A, kanamycin B, neomycin, geneticin (G418), and paromomycin.

**Figure 6:**
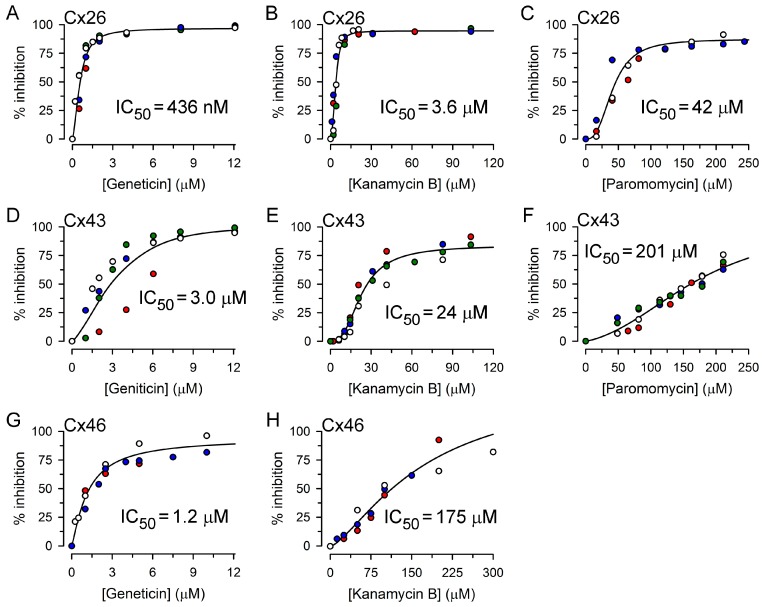
Inhibition of connexin-dependent growth complementation by kanamycin B, geneticin and paromomycin. (**A**–**C**) Effects on Cx26-dependent complementation. (**D**–**F**) Effects on Cx43-dependent complementation. (**G**,**H**) Effects on Cx46-dependent complementation. Each symbol denotes the average of a triplicate measurement, and each color corresponds to an independent experiment. The lines represent fits of Hill’s equation to the data. In all cases, growth inhibition was significant, with a *p* of at least 0.01 vs. growth in the absence of drug.

Kanamycin A, kanamycin B, neomycin, geneticin, and paromomycin inhibited connexin-dependent growth complementation, but the concentrations that produced 50% inhibition (IC_50_) varied by a factor of ~500 between the different isoforms (Table 1). The lowest IC_50_ was for geneticin on Cx26-dependent complementation (~0.4 μM), and the highest was for paromomycin on complementation by Cx43 (~200 μM). The IC_50_ for inhibition of Cx46-dependent growth complementation by paromomycin was not determined, but was even higher than that for inhibition of Cx43 HCs because at 200 μM the inhibition was just 37 ± 4% (*n* = 3). Examples of inhibition of complementation for some of the AGs tested are shown in Figure 6.

## 3. Materials and Methods

### 3.1. Molecular Biology and LB2003 Cells

Details of the human Cx26, Cx43, and Cx46 DNAs used to transform LB2003 cells have been published [22]. The experiments were carried out in accordance with the Texas Tech University Health Sciences Center (TTUHSC) approved guidelines and the experimental protocols were approved by the TTUHSC Recombinant DNA Biosafety Committee. Briefly, pQE60 plasmids containing the connexin DNAs and plasmid pREP4 were used for transformation of *E. coli* LB2003 cells (generously provided by Dr. E.P. Bakker, Osnabrück University, Osnabrück, Germany). The pQE60 plasmids confer resistance to ampicillin, and pREP4 confers resistance to the AGs used in this study. LB2003 cells are missing three key K^+^ transporters (Δtrk, Δkup, and Δkdp strains) and as a result cannot grow in low-[K^+^] media [22,23,25]. Transformed LB2003 cells were stored as a 25% glycerol stock at −80 °C.

### 3.2. Growth Complementation Assay

Details and validation of the growth complementation assay and its optimization for complementation dependent on Cx26 and Cx43 have been published [22]. Basically, freshly prepared competent LB2003 containing the pREP4 plasmid were transformed with pQE60 (empty plasmid; negative control), pQE-Cx26 (human Cx26 DNA into pQE60), pQE-Cx43 (human Cx43 into pQE60) or pQE-Cx46 (human Cx46 into pQE60). After growing overnight in Luria-Bertani (LB) medium (BD, Franklin Lakes, NJ, USA) supplemented with 100 mM KCl (to allow growth) and 0.4 mg/mL ampicillin, the cells were washed four times with NLM, to remove residual potassium from the LB medium, and were resuspended in complementation growth medium to an OD_600_ of 0.2. NLM contained 46 mM Na_2_HPO_4_, 23 mM NaH_2_PO_4_, 8 mM (NH_4_)_2_SO_4_, 0.4 mM MgSO_4_, 0.012 mM FeSO_4_, 1 mM sodium citrate, 44 mM glucose, and 0.006 mM thiamine hydrochloride, pH 7.0. KLM was identical to NLM except for the equimolar replacement of Na^+^ with K^+^. For growth-complementation by Cx26, Cx43, and Cx46 in low-[K^+^] medium we used NLM + 4 mM KCl, NLM + 8 mM KCl, and NLM + 16 mM KCl, respectively. Expression of Cx26 was induced with 0.5 mM IPTG (GoldBio, St. Louis, MO, USA) at the time of dilution to OD_600_ = 0.2. For growth-complementation by Cx43 and Cx46, we used 10 µM IPTG. Transformed cells in the appropriate growth medium were seeded in 24- or 96-well plates, and the plates were incubated at 30 °C, with shaking at 250 and 500 rpm, respectively. OD_600_ was measured after 18 h manually for the 24-well plates or in a plate reader for the 96-well plates (Falcon 353072, Corning Inc., Corning, NY, USA). The initial OD_600_ = 0.2 was subtracted for growth calculations.

### 3.3. Statistics

Data are presented as means ± SEM. Statistically significant differences were calculated by a Student’s *t*-test for unpaired data or one-way ANOVA, as appropriate. Data were obtained from at least three independent experiments, with three repeats per experiment.

## 4. Summary and Conclusions

A role of increased number of open HCs and/or abnormally open HCs (“leaky HCs”) in cell damage has been proposed or demonstrated in disorders of the heart (e.g., cardiac infarction and arrhythmias), the inner ear (deafness), the nervous system (e.g., stroke, oculodentodigital dysplasia, and Charcot-Marie-Tooth disease), the skin (e.g., forms of keratitis, keratoderma, and ectodermal dysplasia), and the eye (cataracts) [8,10,11,13,33,34]. This makes HCs an attractive pharmacological target for inhibition. The results presented here show that AGs of different structures inhibit connexin-dependent complementation of the growth of LB2003 cells in low-[K^+^] medium. We have demonstrated that human connexin HCs expressed in bacteria are functional and that growth complementation of LB2003 cells in low-[K^+^] medium is the result of K^+^ uptake through HCs [21,22]. Here, we show that several AGs inhibit human connexin HCs, expanding previous observations in mammalian cells, frog oocytes, and bacteria.

Advantages and disadvantages of the cell-growth assay used here have been discussed in recent publications [22,35]. An important point to stress is that the assay was not designed to study HC properties but to address the need for a simple test for the discovery of HC inhibitors. It was optimized for simplicity, high signal-to-noise ratio, and reproducibility, with all components added in one step and a simple one-point OD_600_ readout [22,35]. There are well-established assays of HC function that are based on direct measurements of currents or solute transport [5,36]. In contrast, the cell-growth assay is indirect: the readout is a complex phenomenon (cell growth) that results from a consequence of HC function (K^+^ influx). This is an important consideration because K^+^ influx is necessary for cell growth, but the relationship between intracellular [K^+^] and growth is not a simple linear relationship. Another important point is that cell growth associated with expression of HCs (“large” poorly selective channels) depends on the balance between the presence of enough HCs for sufficient K^+^ influx (to promote growth) and not so many active HCs, which will result in unbalanced fluxes (e.g., increased Na^+^ influx, increased efflux of low-molecular weight organic metabolites, intracellular acidification, membrane depolarization, etc.) and decreased growth. In this context, one of the optimization approaches was to change [IPTG] to produce the best signal-to-noise ratio and reproducibility for each isoform [22]. An insufficient amount of IPTG with a low number of HCs results in reduced cell growth, whereas too much IPTG with too many active HCs reduces growth.

K^+^ influx depends on the number of HCs, the single-HC conductance (γ), the open probability (*P_o_*), and the electrochemical K^+^ gradient. Increasing [K^+^] medium increases the driving force for [K^+^] influx, but too much [K^+^] reduces the signal-to-noise ratio by allowing growth in the absence of HCs. Although there are differences between the γ of HCs formed by Cx26, Cx43, and Cx46, the main differences under the conditions of the assays likely reside in the expression (e.g., Cx43 > Cx26) [22], *P_o_*, and transmembrane gradients. The high cell-membrane voltage should reduce *P_o_*, but the low [divalent cation] in the medium (nominally calcium-free; 0.4 mM total magnesium, partly complexed as citrate and phosphate salts) should increase it [37,38]. The predominance of dephosphorylated over phosphorylated connexins in the bacteria would also increase Cx43 HCs *P_o_* [4]. The electrochemical gradients are also potentially different in the assays of different isoforms (differences in ion gradients and membrane voltage due to HC activity), and the [K^+^] medium is only one factor among several. Since the isoform-specific assay conditions are unique, differences in the [IPTG] and [K^+^] cannot be used to conclude about the properties of the HCs expressed in bacteria; they were empirical tools, modified to produce the best assay. In summary, the cell-growth assay is well-suited to address comparative inhibition of HCs by AGs; although it is important to understand its limitations, it is also important to emphasize its simplicity, low cost, high sensitivity, excellent reproducibility, and scalability for high-throughput screening.

The inhibition of Cx26-dependent growth complementation occurred at lower concentrations for all AGs vs. Cx43 and Cx46. The pattern of isoform selectivity was best for kanamycin B, with IC_50_ ratios relative to Cx26 of ~7 for Cx43 and ~50 for Cx46. Ototoxicity and nephrotoxicity may occur at the concentrations necessary to efficiently inhibit HCs, especially Cx43 and Cx46 HCs. However, the major drive for our studies was to identify commercial AGs that can serve as suitable lead compounds for the synthesis of derivatives with improved properties (higher affinity and selectivity, absence of antibiotic and toxic effects). Considering that kanamycins and neomycin have been used in humans, these AGs and geneticin, which displays the lower IC_50_, seem to be good lead candidates for the development of new connexin HC inhibitors.

## Figures and Tables

**Table 1:** Aminoglycoside concentration needed to inhibit growth complementation by 50% (IC_50_).

Aminoglycoside	Growth Complementation Dependent on		
	Cx26	Cx43	Cx46
Kanamycin A	11.5 ± 1.8 μM (3)	48 ± 2 μM (4)	112 ± 5 μM (3)
Kanamycin B	3.6 ± 0.9 μM (4)	24 ± 2 μM (4)	175 ± 9 μM (3)
Geneticin	0.44 ± 0.06 μM (5)	3.0 ± 0.8 μM (4)	1.2 ± 0.2 μM (3)
Neomycin	7.4 ± 1.3 μM (5)	45 ± 9 μM (4)	16 ± 2 μM (3)
Paromomycin	42 ± 9 μM (3)	201 ± 17 μM (4)	>200 μM (3)

IC_50_s were calculated from fittings of Hill’s equation to the data. For each group, means ± standard error (SEM) were calculated from triplicate averages of 3–5 independent experiments (in parenthesis). The inhibition of growth complementation was essentially complete (93 ± 3%). Hill coefficients were not statistically different between groups, with a mean ± SEM of 2.1 ± 0.2.
